# Scalp Microbiome Composition in Young Women: Associations with Scalp Type, Sensitivity, and Lifestyle Factors

**DOI:** 10.3390/life16010091

**Published:** 2026-01-07

**Authors:** Ying Guo, Yao Zhang, Qiaoni Hui, Shenshen Zhu, Jingtao Wang, Liya Song

**Affiliations:** 1 Department of Cosmetics, School of Light Industry Science and Engineering, Beijing Technology and Busines University, Beijing 100048, China; gy11072000@163.com (Y.G.); jiajingwenzhangyao@126.com (Y.Z.); huiqiaoni1111@163.com (Q.H.); zhuss2024@163.com (S.Z.); 18295503378@163.com (J.W.); 2 Beijing Key Lab of Plant Resources Research and Development, Beijing Technology and Business University, No. 11 Fucheng Road, Haidian District, Beijing 100048, China

**Keywords:** scalp microbiome, microbial community structure, scalp type, sensitive scalp, lifestyle, psychological stress, interaction

## Abstract

Background: The scalp represents a distinct ecological niche within the skin, and the structure of its microbiota, together with the factors shaping it, is considered important for the maintenance of scalp health. Methods: This study systematically analyzed the bacterial and fungal community structures on the scalps of 63 healthy Chinese women aged 18–25, and examined their associations with scalp type, sensitivity, and lifestyle factors. Scalp samples were collected, questionnaire surveys were administered, scalp physiological parameters were measured, and high-throughput sequencing of 16S rRNA and ITS genes was performed. Results: The results showed that, in this unique scalp skin niche, the dominant bacterial phylum was Actinobacteria, while the dominant fungal phylum was Ascomycota. The predominant bacterial genera were *Cutibacterium* and *Staphylococcus*, and the fungal community was dominated by *Malassezia*. When scalp types were categorized according to sebum content, dry scalps showed enrichment of *Micrococcus*, *Streptococcus, Delftia*, *Staphylococcus aureus*, and *Staphylococcus hominis*, whereas oily scalps, on the other hand, are primarily colonized by *Cutibacterium* and *Staphylococcus species*. In addition, we observed microbial interactions under different physiological conditions. The relative abundance of *Cutibacterium* decreased with increasing scalp sensitivity. Higher psychological stress, insufficient sleep, and high-sugar/high-fat dietary patterns tended to coincide with shifts in the relative abundance of *Malassezia*, implying that these influences may act through fungal rather than bacterial components of the scalp microbiota. Scalp sensitivity showed the strongest association with β-diversity among the variables examined, although the effect size was modest and did not reach conventional significance in the multivariable PERMANOVA. Conclusions: In young women, the scalp constitutes a distinct cutaneous niche whose microbiota is jointly shaped by sebum level, barrier sensitivity, and lifestyle factors, with sensitivity emerging as one of the more influential dimensions of community variation. These findings provide guidance for future in-depth research on the scalp microbiome network and offer a foundational reference for preventing suboptimal and pathological scalp conditions.

## 1. Introduction

In recent years, increasing attention has been paid to scalp health. The scalp, with its dense hair coverage, abundant sweat and sebaceous glands, and relatively high moisture level, forms a distinctive ecological niche that favors microbial colonization and growth. It is extensively colonized by microorganisms, with an estimated density of 10^3^–10^5^ microorganisms per mm^2^ [1], primarily comprising *Staphylococcus*, *Cutibacterium* and *Malassezia* species [2]. These microorganisms interact not only with each other—through competition, symbiosis, mutualism and parasitism—but also with various components of the scalp environment, thereby maintaining a characteristic dynamic equilibrium [3]. Under healthy conditions, scalp microbiota contribute to maintaining the functional integrity of the scalp’s physicochemical barrier (including the outer epidermis and its surface chemical defense mechanisms) and help regulate homeostasis within the skin microenvironment. For example, Almoughrabie et al. [4] reported that the lipid-rich environment of the scalp provides nutrients for *Cutibacterium*, which utilize lipids to produce short-chain fatty acids. These metabolites induce keratinocytes to upregulate lipid synthesis, including triglycerides and ceramides, which are key lipid components that play essential roles in maintaining skin barrier integrity. Moreover, propionic acid produced by *Cutibacterium* activates PPARα receptors in keratinocytes, thereby upregulating lipid synthesis-related genes (such as GPAT3) and promoting lipid accumulation, which in turn further reinforces the lipid-enriched microenvironment. Furthermore, beneficial microbial communities restrict the colonization of potentially harmful microorganisms through competitive exclusion or positional advantage [5,6]. These commensal microbes can also activate immune responses in the scalp, strengthening its defensive capacity and reducing the likelihood of inflammation and infection [5,7]. More recently, *Malassezia globosa* was shown to stimulate keratinocyte IL-23 secretion via the TLR2/MyD88/NF-κB axis, thereby promoting downstream Th17-associated cytokine programs (e.g., IL-17A/IL-22) in the skin [8]. In addition, tryptophan-derived metabolites produced by skin commensals can serve as ligands for the aryl hydrocarbon receptor (AhR), a central regulator of epidermal differentiation and barrier restoration, and may shape inflammatory responses—such as reducing TSLP induction and limiting inflammasome activation—in an AhR-dependent manner [9,10,11]. Importantly, these barrier functions are not independent; they engage in bidirectional cross-talk and coordinated regulation, collectively supporting scalp homeostasis and overall health. However, scalp health may be affected by physiological characteristics such as body mass index (BMI), sebum secretion [12], hair length [13], hormonal levels [14], scalp barrier function [12], and psychological and general health status, as well as daily hair care practices and lifestyle factors including shampoo functionality, washing frequency and the frequency of chemical treatments [15]. Environmental exposures, including ultraviolet radiation and air pollution [16,17], living environment and climatic conditions [18], also can influence scalp health. For example, air pollution (e.g., particulate matter and PAHs) can promote oxidative stress and impair barrier function, and has been linked to reduced microbial diversity and a shift toward taxa associated with changes in TEWL and skin hydration [19,20]. In addition, UV radiation may reshape the microbial habitat by increasing oxidative stress (ROS), altering the local immune milieu, and stimulating cutaneous antimicrobial defenses, including the induction of antimicrobial peptide (AMP) expression in keratinocytes [21].

Progress in high-throughput sequencing has substantially advanced our understanding of the scalp microbiome. The scalp/hair follicle represents a distinct, lipid-rich niche with unique microbial ecology and host interactions [22]. In this niche, growing evidence suggests that microbial effects are highly context dependent, shaped by the local environment, including lipid availability and composition, interactions with co-resident commensals, and the status of the host immune and barrier defenses. Together, these factors may determine whether microbes contribute to cutaneous homeostasis or, under certain conditions, promote inflammation. For example, under homeostatic conditions, the skin-resident fungus *Malassezia* secretes the protease MgSAP1, which rapidly hydrolyzes *S. aureus* Protein A and inhibits biofilm formation [23]. When this equilibrium is disrupted, scalp disorders such as dandruff, seborrheic dermatitis, alopecia areata, folliculitis, and scalp psoriasis may arise [3]. As observed in atopic dermatitis, the protease Mfsap1 secreted by *Malassezia* can exacerbate cutaneous inflammation [24]. *Malassezia* fungi mediate sebum peroxidation, leading to the production of squalene monohydroperoxide and malondialdehyde; these metabolites act on epidermal cells and constitute one of the triggers of dandruff [25]. In scalp psoriasis lesions, the relative abundance of *Staphylococcus aureus* increases, whereas that of *Cutibacterium* decreases [26].

Recent reviews increasingly characterize the skin microbiome as a functional ecosystem shaped by host–microbe interactions. However, most systematic investigations have centered on pathological conditions, whereas evidence regarding the non-pathological scalp microbiota remains comparatively limited [27]. Accordingly, this study investigates the compositional characteristics of the young female scalp microbiota (bacteria and fungi) and their relationships with scalp health issues. By performing multidimensional analyses of volunteers from multiple taxonomic perspectives, we further explore the internal and external factors that influence the scalp microbiome.

## 2. Materials and Methods

### 2.1. Subject Recruitment and Sample Collection

A total of 63 healthy Chinese females aged 18–25 years were recruited as volunteers in Beijing. Participants were screened based on the inclusion/exclusion criteria and enrolled consecutively until the target sample size was achieved. Participants were screened using a questionnaire to exclude those with chronic diseases and scalp conditions such as seborrheic dermatitis, psoriasis, pathological hair loss, and folliculitis. Participants had not used any systemic or topical therapeutic agents with the potential to alter sebum production, inflammatory status, or the scalp microbiome within the preceding 6 months (e.g., isotretinoin; antibiotics or antifungals; corticosteroids; keratolytics; or hair-growth medications, etc.). And individuals were excluded if they reported any scalp disease or were receiving scalp-related treatment, or if they had undergone any scalp-focused cosmetic or medical procedures within the previous 6 months (e.g., needle mesotherapy, microneedling, laser/light-based treatments, or chemical exfoliation such as scalp peels). We also excluded participants who had recently permed or dyed their hair, or had other chemical or physical manipulations involving the scalp. In addition, conditioner use was documented; all participants reported applying conditioner only to the mid-lengths and ends of the hair, not directly to the scalp. A questionnaire was used to assess scalp condition, lifestyle habits, psychological factors, prior treatment history, and family history. Participants were asked to avoid washing or otherwise treating their scalps for 48 h before sampling. Scalp specimens were then obtained by investigators wearing sterile disposable gloves, with a new pair used for each individual participant. Sterile cotton swabs moistened with wetting solution ( sterile 0.9% (*w*/*v*) saline containing Tween 20; the solution had a pH close to neutral) were used to sample three 0.6 × 10 cm^2^ areas along hair partings in the crown region by rotating the swab. Each swab was subsequently transferred to a collection tube, and the three swabs obtained from a given participant were pooled for DNA extraction. In parallel, three blank swabs were exposed to air for the same duration and processed identically as negative controls. After collection, all samples were immediately placed on dry ice and subsequently transferred to −80 °C for storage until DNA extraction. All subjects provided informed consent prior to participation. The research protocol was approved by the Ethics Committee for Scientific Research at Beijing Technology and Business University (Ethics No. 244, 2024).

### 2.2. Skin Parameter Measurement

Following microbial sample collection, all scalp biophysical parameters (TEWL, sebum, and surface pH) were measured prior to hair washing under standardized environmental conditions. Scalp parameters were measured in the mirror-image region corresponding to the sampling site, located slightly to the right of the midline at the crown. Transepidermal water loss (TEWL) was recorded as an indicator of skin hydration with a Tewameter TM300 (Courage and Khazaka, Cologne, Germany), and the readings were reported in g/h·m^2^. Sebum production was quantified using a Sebumeter SM815 (Courage and Khazaka, Cologne, Germany), while scalp surface pH was assessed with a skin pH meter (Courage and Khazaka, Cologne, Germany).

### 2.3. DNA Extraction

Total DNA was extracted from the samples using a commercial DNeasy Blood and Tissue Kit (QIAGEN, Hilden, Germany). The extracted DNA was stored at −80 °C until further analysis.

### 2.4. MiSeq Sequencing Workflow

The sequencing workflow consisted of PCR amplification of target regions, quantitative fluorescence analysis, paired-end (PE) library construction, and Illumina MiSeq sequencing. For bacterial profiling, the V1–V3 variable regions of the 16S rRNA gene were amplified using the primer pair 27F–533R, including the forward primer 27F (5′-AGAGTTTGATCMTGGCTCAG-3′) and the reverse primer 533R (5′-TTACCGCGGCTGCTGGCAC-3′). For fungal profiling, the internal transcribed spacer (ITS) region was amplified by PCR using primers ITS1F (5′-CTTGGTCATTTAGAGGAAGTAA-3′) and ITS2R (5′-GCTGCGTTCTTCATCGATGC-3′). Paired-end reads obtained from Illumina sequencing were first merged based on their overlap and subjected to quality control and filtering; after assigning reads to each sample, OTU (operational taxonomic unit) clustering analysis and taxonomic classification were performed, and OTUs were then used for clustering and statistical evaluation of community composition across all taxonomic ranks.

### 2.5. Skin Microbiome and Microbial Community Analysis

We used UPARSE (v7.0.1090) [28] to cluster the sequences into operational taxonomic units (OTUs) based on a 97% similarity threshold. Taxonomic annotation of the OTU representative sequences was then carried out with the SILVA v138.2 reference database using the RDP Classifier [29]. Sequences annotated as mitochondrial or chloroplast origin were removed from the dataset. Alpha diversity metrics (Chao and Shannon) were computed using the mothur (1.30.2) [30] software package. Differences in alpha diversity between groups were assessed with the Wilcoxon rank-sum test. Principal coordinates analysis (PCoA) based on Bray–Curtis dissimilarities was then applied to evaluate and visualize similarities in microbial community structure across samples. In combination with the nonparametric PERMANOVA test, this approach was used to determine whether differences in community structure between sample groups were statistically significant. LEfSe (Linear Discriminant Analysis Effect Size) [31] (LDA > 2, *p* < 0.05) was applied to identify bacterial taxa with significantly different relative abundances between groups from the phylum to the genus level.

### 2.6. Microbial Network Analysis

To construct the microbial network, the top 20 bacterial and fungal genera ranked by relative abundance were selected. Spearman’s correlation analysis was used to calculate symbiotic (positive) and competitive (negative) relationships among microorganisms. Correlation pairs with *p* < 0.05 and an absolute correlation coefficient > 0.5 were retained for network construction. The NetworkX package was then used to build the co-occurrence network and to calculate node degree distribution, network diameter, average shortest path length, node connectivity, closeness centrality, betweenness centrality, and other relevant metrics for visualization. Spearman correlation heatmaps were generated to explore associations between bacterial genera and environmental variables, using the pheatmap package in R (v3.3.1). To evaluate scalp microbiome β-diversity, we applied a multivariable PERMANOVA (adonis function, vegan package in R) to Bray–Curtis distance matrices derived from genus-level community profiles. Scalp type (dry, medium, oily), barrier sensitivity (mild, moderate, severe), and lifestyle variables (sleep quality, high-sugar diet, and perceived stress) were entered together as fixed-effect predictors in a single model, and permutations were stratified by individual to appropriately account for repeated samples when present.

## 3. Results

### 3.1. Scalp Characteristics of Participants

Through questionnaire screening, individuals with scalp diseases were excluded. A total of 63 subjects aged 18–25 years were subsequently selected for microbial sampling and measurement of scalp physiological parameters. On the basis of sebum measurements, subjects were classified into three groups: oily group (*n* = 25; sebum content > 150 μg/cm^2^), dry group (*n* = 18; sebum content < 40 μg/cm^2^), and medium group (*n* = 20; sebum content 70–120 μg/cm^2^). According to Sensitive 3S questionnaire scores, subjects were categorized as non-sensitive (*n* = 12), mildly sensitive (*n* = 9), sensitive (*n* = 25), and severely sensitive (*n* = 14). Oily scalp was determined using both sebum meter readings on the scalp and self-assessment. A sebum meter was used to measure sebum content at the vertex, together with self-assessment by the subjects; according to the criteria that oily subjects had sebum levels greater than 150 μg/cm^2^ and dry subjects had sebum levels less than 40 μg/cm^2^ [32], a dry-sensitive group (Dry_Min, *n* = 14) and an oily-sensitive group (Oil_Min, *n* = 20) were obtained. Subgroup analyses were further conducted according to self-reported stress levels, sleep quality, and dietary habits.

### 3.2. Results of the Scalp Microbiome in Young Women

Following quality control and filtering of the raw high-throughput sequencing data, valid sequences were retained for downstream analyses.

For bacteria, a total of 3,935,095 high-quality sequences were obtained from 64 samples, including 63 scalp samples and 1 negative control sample, with an average read length of 474 bp. At 97% sequence similarity, these sequences were clustered and taxonomically annotated, yielding 661 OTUs, which were assigned to 17 bacterial phyla, 291 genera and 453 species.

For fungi, diversity analysis was completed for 64 samples, resulting in 3,919,908 sequences with an average read length of 259 bp. At 97% sequence similarity, clustering and taxonomic annotation identified 1198 OTUs, which were assigned to 11 fungal phyla, 484 genera and 781 species.

**Figure 1:**
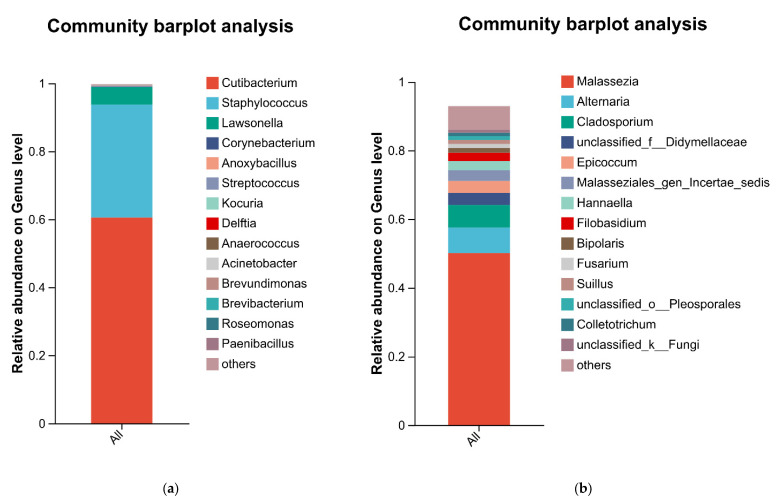
Sample (**a**) and fungal (**b**) composition at the genus level.

Analysis of the scalp bacterial community composition showed that the bacterial phyla Actinomycetota and Bacillota predominated on the scalp (Table 1), together accounting for more than 95% of the total bacterial relative abundance. At the genus level, the dominant genera were *Cutibacterium*, *Staphylococcus*, and *Lawsonella*, (Figure 1) which together represented more than 95% of the bacterial relative abundance. On the scalp, fungi were mainly distributed within the phyla Basidiomycota and Ascomycota, with *Malassezia*, *Cladosporium*, and *Alternaria* as the predominant genera.

### 3.3. Microbial Composition and Differences Among Young Female Participants with Different Scalp Types

#### 3.3.1. Microbial Composition and Differences Among Dry, Medium, and Oily Scalp Groups

Analysis of alpha-diversity indices for samples from the dry (*n* = 18), oily (*n* = 25), and medium (*n* = 20) scalp groups showed no statistically significant intergroup differences in either bacterial or fungal diversity. Similarly, OTU richness (Chao and Sobs indices) and the Shannon diversity index did not differ significantly among the three groups. Principal coordinates analysis (PCoA) based on Bray–Curtis distances showed no clear separation of bacterial or fungal communities among the three groups.

**Figure 2:**
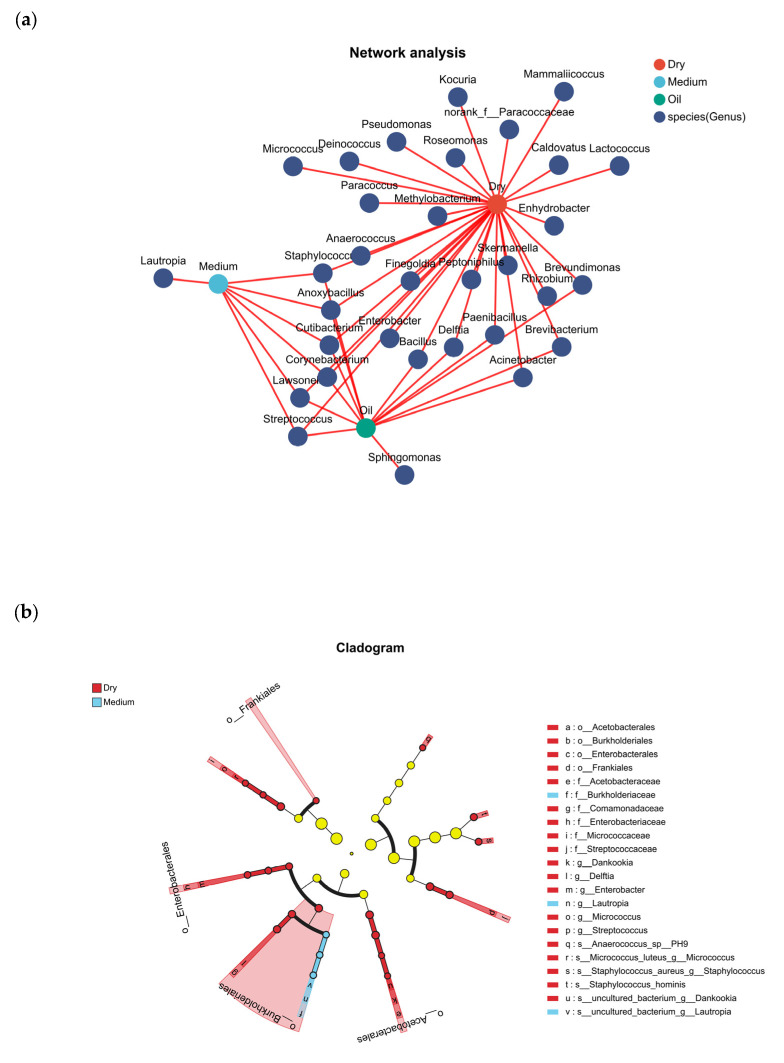
Co-occurrence network and LEfSe cladogram. (**a**) Co-occurrence network showing species relationships in different samples. Nodes represent samples or species, and edges indicate that a species is present in a given sample. (**b**) LEfSe cladogram of taxa that are enriched in the three scalp-type groups.

Co-occurrence network and LEfSe analyses (Figure 2) showed that *Cutibacterium*, *Staphylococcus*, *Lawsonella*, *Corynebacterium*, *Streptococcus* and *Anoxybacillus* were shared among the microbiomes of all three scalp types.

Significance tests at the genus level were performed among the dry group (Dry), oily group (Oil) and medium group (Medium). No significant differences were observed at the phylum level for Actinomycetota and Bacillota among the three groups. At the genus level, there were no significant differences in the dominant genera ranked by relative abundance, namely *Cutibacterium*, *Staphylococcus* and *Lawsonella*. Instead, differences were concentrated in genera with lower relative abundances, including *Anoxybacillus* (*p* = 0.03708), *Streptococcus* (*p* = 0.03213) and *Kocuria* (*p* = 0.04392), all of which were significantly enriched in the dry scalp group. At the species level, compared with the oily and medium scalp groups, the dry scalp group showed significantly higher abundances of *Staphylococcus aureus*, *Staphylococcus epidermidis* and an unclassified *Staphylococcus species* (*Staphylococcus sp. M0911*) within the genus *Staphylococcus*. In addition, analysis of the fungal community composition revealed no significant differences in dominant genera at either the phylum or genus levels among the three groups.

#### 3.3.2. Correlation Network Analysis Among Dry, Medium, and Oily Scalp Groups

**Figure 3:**
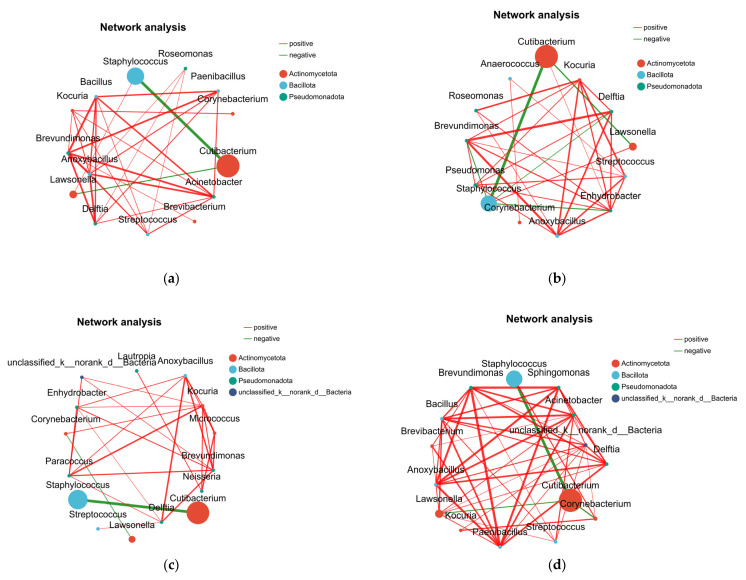
Univariate correlation networks of bacterial communities for different scalp types. (**a**) All samples; (**b**) dry scalp; (**c**) medium scalp; (**d**) oily scalp.

Correlation analysis was performed on bacterial species with relatively high abundance across all scalp samples, using a screening threshold of |r| ≥ 0.4 and *p* < 0.05. As shown in the figure, 19 significantly correlated species were identified (Figure 3). Co-occurrence analysis of bacterial communities on dry, medium, and oily scalps showed that similar bacterial clusters appeared in all three groups, but the strengths of their correlations differed. In all scalp types, *Cutibacterium* and *Staphylococcus* consistently exhibited a strong negative correlation. Although the overall community composition varied, each scalp type displayed tightly connected and strongly correlated relationships among certain taxa.

In the dry scalp environment, *Staphylococcus* showed negative correlations with *Brevundimonas*, *Delftia*, *Enhydrobacter*, *Caldovatus*, and *Cutibacterium*, but a positive correlation with *Corynebacterium*. In the oily scalp environment, *Cutibacterium* was negatively correlated with *Staphylococcus*, *Corynebacterium*, and *Lawsonella*. These findings may reflect different patterns of microbial interactions under distinct scalp conditions, or may result from differences in community composition between groups; the specific mechanisms require further experimental validation.

**Figure 4:**
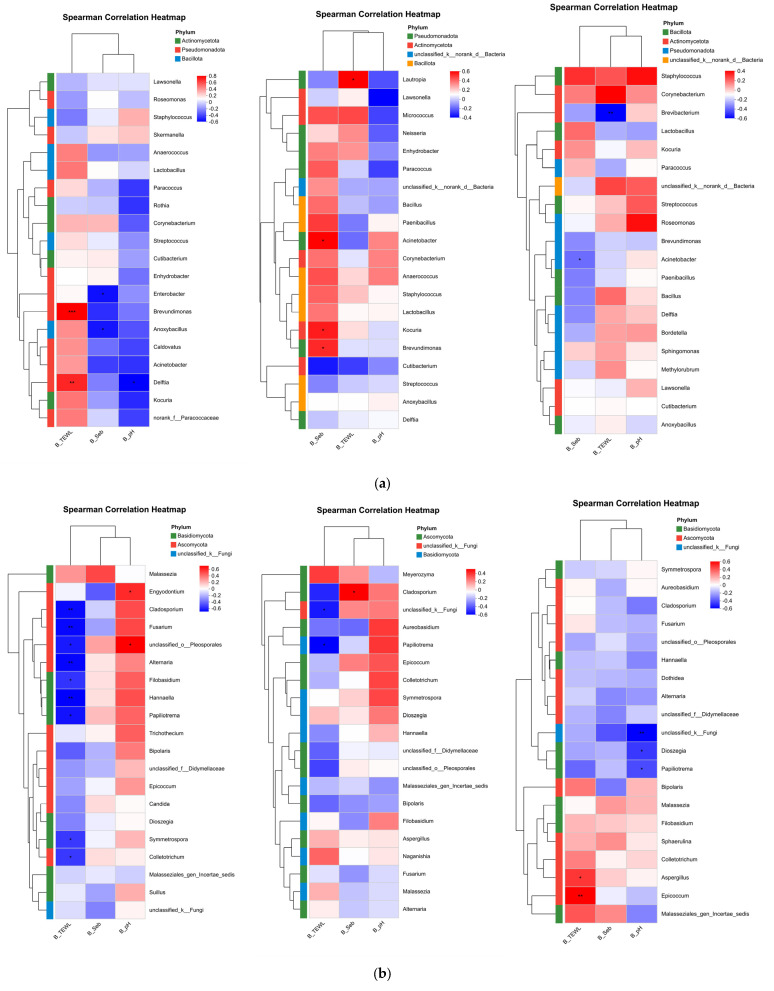
Univariate Correlation Network Diagram; Bacterial genera (**a**), Fungal genera (**b**); from left to right: Dry group, Medium group, Oil group; The *X*−axis and *Y*−axis represent environmental factors and species, respectively. Correlation R values and *p* values were calculated. R values are displayed in different colors in the figure. *p* values less than 0.05 are marked with an asterisk (*). The legend on the right shows the color ranges for different R values. * 0.01 < *p* ≤ 0.05, ** 0.001 < *p* ≤ 0.01, *** *p* ≤ 0.001. Where B_TEWL: Trans Epidermal Water Loss before shampooing; B_Seb: Sebum content before shampooing; B_pH: Scalp pH before shampooing.

Correlation heatmaps were used to assess the influence of different environmental factors on scalp bacterial communities at the genus level (Figure 4). In the oily scalp group, most bacterial genera showed a negative correlation with sebum levels, with Acinetobacter displaying a statistically significant negative correlation. This may indicate that a high-sebum environment inhibits the growth of certain microorganisms. In the dry scalp group, most bacterial genera were negatively correlated with pH and positively correlated with transepidermal water loss (TEWL). Notably, *Delftia* showed a significant negative correlation with pH and a significant positive correlation with TEWL.

Correlation heatmap analysis of the fungal communities showed that, in dry scalps, several fungal genera were negatively correlated with TEWL. In particular, *Alternaria*, *Cladosporium*, *Hannaella*, *Filobasidium* and *Fusarium* exhibited significant negative correlations with TEWL values (*p* < 0.05). However, fungal genera overall did not display strong correlations with sebum levels, suggesting a more complex relationship between fungi and sebum production.

#### 3.3.3. Microbial Composition and Differences in Sensitive Populations

**Figure 5:**
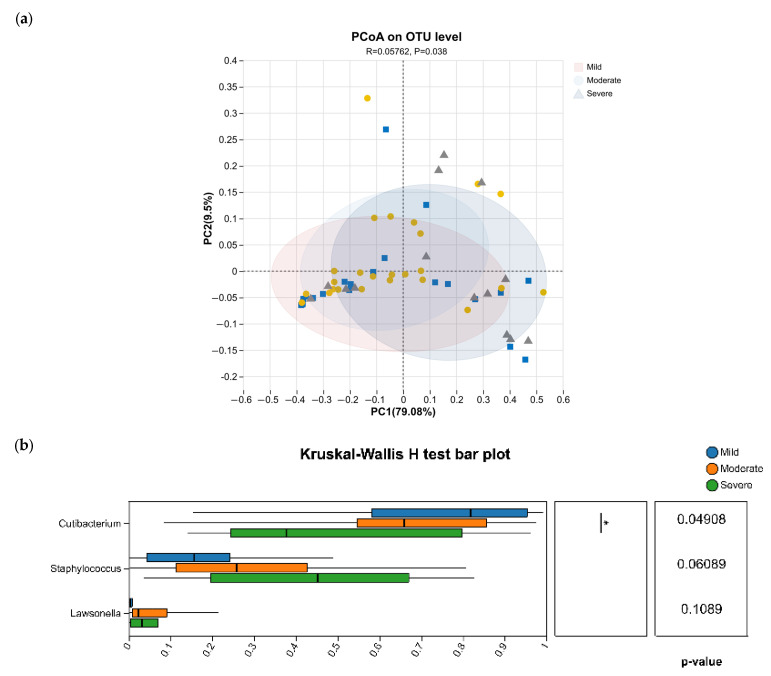
PCoA and bacterial differences among scalp sensitivity groups. (**a**) PCoA plots of bacterial communities in the mild, normal and severe sensitivity groups. (**b**) Bacterial taxa that differ among the three sensitivity groups. * 0.01 < *p* ≤ 0.05.

According to the Sensitive 3S questionnaire scores, subjects were classified into mild sensitivity (n = 9), sensitivity (n = 25), and severe sensitivity (n = 14) groups. Analysis of microbial community diversity indices showed no significant differences in bacterial or fungal community abundance or in alpha-diversity indices (Chao, Sobs, Shannon index) among the three groups. Principal coordinate analysis (PCoA) revealed clear separation of bacterial community structures among the three groups, whereas no obvious separation was observed for fungal communities (Figure 5).

In the analysis of bacterial taxa differing among the mildly sensitive, sensitive, and severely sensitive scalp groups, we found that at the genus level, the relative abundance of *Cutibacterium* decreased progressively with increasing sensitivity (*p* = 0.04908), indicating a significant negative association between *Cutibacterium* abundance and sensitivity level. By contrast, the relative abundance of the genus *Staphylococcus* tended to increase with increasing sensitivity (*p* = 0.06089). Other differentially abundant genera were predominantly those with relatively low abundance. Analysis of fungal taxa did not reveal similar gradient changes.

Based on sebum measurement parameters and 3S sensitivity questionnaire scores, subjects were further stratified into two subgroups: Dry_Min (*n* = 14; dry scalp with sensitivity) and Oil_Min (*n* = 20; oily scalp with sensitivity).

**Figure 6:**
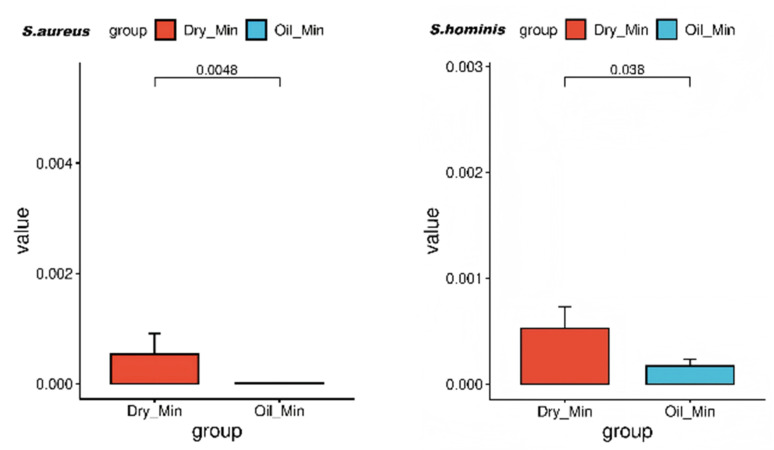
Bacterial species-level differences between Dry_Min and Oil_Min.

In the intergroup comparison of bacterial genera between the Dry_Min and Oil_Min groups, no significant differences were found in the dominant genera *Cutibacterium*, *Staphylococcus* and *Lawsonella*. However, at the species level, the relative abundances of *Staphylococcus hominis* and *Staphylococcus aureus* were significantly lower in the oily-sensitive group than in the dry-sensitive group (Figure 6). As several subgroups included relatively few participants, the results should be validated in future studies involving larger cohorts.

### 3.4. Effects of Different Living Conditions and Psychological Stress on Scalp Microbiome Composition

Based on questionnaire responses, we divided the subjects into a no-stress group (UN, *n* = 3), a mild-stress group (General, *n* = 47), and a high-stress group (High, *n* = 10); into a non–high-sugar/high-fat diet group (U_Sugar, *n* = 38) and a high-sugar/high-fat diet group (H_Sugar, *n* = 22); and into a group with sleep duration > 6 h (U_Late, *n* = 37) and a group with sleep duration < 6 h (Late, *n* = 23).

**Figure 7:**
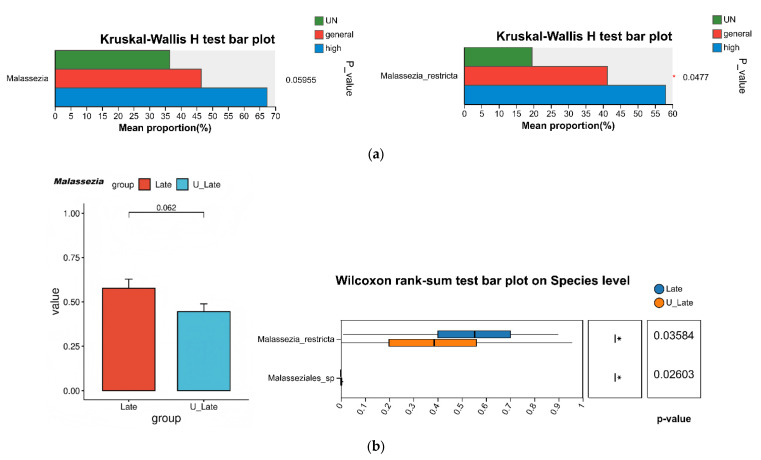
Differences in *Malassezia* and *Malassezia restricta* composition across stress levels (**a**); Genus- and species-level differences between medium and insufficient sleep groups (**b**). * 0.01 < *p* ≤ 0.05.

When samples were grouped according to psychological stress level, dietary pattern and sleep quality, no significant differences were observed in the richness or diversity of bacterial and fungal communities. However, intergroup comparison revealed that these lifestyle and mental health factors did not result in marked changes in bacterial community composition, but were more evident in fungal community composition, manifesting mainly as shifts in the relative abundances of fungal genera. We observed that with increasing psychological stress, the genus *Malassezia* and its most abundant species, *Malassezia restricta*, both showed an upward trend in relative abundance (*p* = 0.06542; *p* = 0.05089), which may suggest a potential positive association between stress level and *Malassezia* abundance (Figure 7). In addition, individuals who reported a preference for high-sugar, high-fat diets exhibited higher colonization by *Malassezia* in their fungal communities. Differences at the genus level were also observed between participants with sleep duration > 6 h and those with <6 h, with shorter sleep duration showing a trend toward a higher relative abundance of *Malassezia* (*p* = 0.08282). At the species level, *Malassezia restricta* was nominally more abundant in the insufficient-sleep group than in the group sleeping more than 6 h (*p* = 0.04802). Given the small number of participants in the stress-based groups, these observations need to be corroborated in studies that include larger sample sizes. Furthermore, several of the aforementioned associations did not remain statistically significant after correction for multiple testing; therefore, we interpret them as exploratory signals that may indicate potential trends rather than definitive effects.

### 3.5. Sensitivity as a Prominent Dimension in Scalp Microbial Differences

Using multifactor PERMANOVA, we found that the variables included in the model collectively explained 14.4% of the variation in scalp microbial β-diversity among young women (Table 2). Among the physiological and barrier-related indicators, scalp sensitivity showed the stronger association with community structure (R^2^ = 0.105, *p* = 0.083), while lifestyle-related measures—sebum content, transepidermal water loss (TEWL), sleep, and dietary pattern—each accounted for additional but relatively smaller portions of the variance.

## 4. Discussion

Most studies on the scalp microbiome have focused on disease states, whereas relatively little attention has been paid to the characteristics of the clinically normal scalp. Therefore, we systematically characterized the scalp microbiota of young women under non-pathological conditions and examined the factors associated with its composition. In this study, we found that the predominant bacterial phyla on the scalp were Actinobacteria and Bacillota, while fungi were mainly assigned to Basidiomycota and Ascomycota, despite substantial interindividual variation. At the genus level, *Cutibacterium* and *Staphylococcus* were the dominant bacterial genera, and *Malassezia* was the dominant fungal genus. These findings are consistent with the results of Rituja Saxena et al. [2] who investigated the scalp microbiota of 70 Indian women.

At the same time, we found that the scalp microbiota has a distinct composition compared with other skin sites, including facial skin, which is also exposed to the external environment. Our previous work in women of the same age group [33] showed that, at the genus level, the facial bacterial community of young women is dominated by *Cutibacterium* and *Staphylococcus*, which together account for about 60% [34] of the total bacterial abundance, while the remainder consists is composed of a series of genera present at relatively low abundances. This pattern differs markedly from that on the scalp, where the dominant bacterial genera are also mainly *Cutibacterium* and *Staphylococcus*, but together they represent more than 90% [35] of the bacterial community. The facial fungal community is mainly composed of *Aspergillus*, *Cladosporium*, *Candida*, *Cryptococcus*, *Malassezia*, and other genera, whereas on the scalp *Malassezia* is predominant. Thus, the scalp bacterial community appears more concentrated, while the facial fungal community shows higher diversity and a more dispersed composition. These differences may be related to the distinct ecological environments of the two sites, particularly sebum secretion. In young women with oily facial skin, sebum secretion is approximately 24.94 μg/cm^2^, which is much lower than the sebum level on the scalp 115 μg/cm^2^. These differences may be closely related to the unique ecological niche of the scalp [36]. The invaginated hair follicle–sebaceous gland units provide a microenvironment favoring colonization by obligate and facultative anaerobes, and the abundant secretions from sweat and sebaceous glands supply nutrients for lipophilic microorganisms [37]. Dense hair coverage helps retain moisture, forming a humid, slightly acidic microenvironment that further promotes *Staphylococcus* colonization [37,38]. In addition, interactions among microorganisms and between microorganisms and the host likely contribute to the observed compositional differences. *Malassezia* has lost the ability to synthesize fatty acids and metabolize carbohydrates de novo, rendering it highly dependent on exogenous lipids from host sebum for survival. Its genome encodes multiple lipases capable of hydrolyzing triglycerides and polyunsaturated fatty acids secreted by scalp sebaceous glands, thereby providing nutrients for its own growth [39]. Nevertheless, in the present study we did not observe significant differences in dominant bacterial genera among dry, oily and medium scalp types. One explanation may be that sebum alone does not fully reflect scalp condition; rather, the complex scalp niche is shaped by numerous interacting factors, so the effects of sebum on the scalp may not be as pronounced as on other skin sites [40].

Scalp sensitivity is one of the most common manifestations of sensitive skin syndrome. Patients typically experience itching, stinging, tightness, pain and burning in response to external triggers, while showing no clear clinical evidence of inflammation, and this condition has not yet been formally recognized as a separate dermatologic disorder. In our study, no significant differences were detected in the dominant scalp bacterial genera between non-sensitive and sensitive individuals. However, we observed that the relative abundance of *Cutibacterium* decreased with increasing sensitivity scores on the Sensitive 3S questionnaire, whereas *Staphylococcus* showed the opposite trend. Farage et al. [41] summarized existing work on sensitive skin and reported that individuals with sensitive skin tend to have reduced sebum and hydration levels. Clinical data further indicate impaired epidermal barrier function and increased Transepidermal Water Loss in these individuals [42]. This may worsen the growth conditions for *Cutibacterium* and contribute to its reduced abundance. *Cutibacterium* can activate the PPARα–GPAT3 pathway by producing propionic acid, thereby inducing lipid synthesis. The accumulation of lipids improves intercellular junctions, reduces water loss and limits the penetration of exogenous substances [4], ultimately strengthening the barrier. A decline in *Cutibacterium* abundance may therefore further compromise the barrier and help to sustain a feedback loop of barrier impairment. In parallel, impairment of the skin barrier leads to increased pH, which creates a more favorable environment for *Staphylococcal* proliferation [43,44]. This pattern may also be linked to alterations in host immune regulation. Thus, the antagonistic relationship between *Cutibacterium* and *Staphylococcus* may intensify with increasing sensitivity: *Cutibacterium* declines, and *Staphylococcus* expands under reduced competitive pressure.

In this study, we not only examined the effects of sebum secretion and scalp sensitivity on microbial composition, but also assessed the impact of lifestyle factors and psychological stress on the scalp microbiota. Our findings indicate a potential positive association between stress level and the relative abundance of *Malassezia*, and shorter sleep duration showed a similar relationship with *Malassezia* colonization. One possible explanation is that chronic stress activates the hypothalamic–pituitary–adrenal (HPA) axis, leading to elevated cortisol levels [45], which in turn can promote sebum secretion. Because *Malassezia* primarily utilizes lipids in sebum as a nutritional source, increased sebum production may provide a more favorable environment for its proliferation. In addition, stress-induced increases in cortisol can suppress Th1-type immune responses and weaken host immune surveillance against fungi [46]. Insufficient sleep may further disturb the medium repair cycle of the skin barrier, increase epidermal permeability and create physical conditions that facilitate fungal colonization [47]. Sleep deprivation also impairs immune function and exacerbates inflammatory responses [48]; for example, it can alter the balance of Th1-associated chemokines and reduce CD4^+^ T-cell levels, thereby affecting immune homeostasis [49] and influencing *Malassezia* colonization.

Several limitations should be acknowledged. First, after further stratification by scalp type, sensitivity level, lifestyle factors, or psychological stress, some subgroups included relatively few participants. As a result, the observed associations may be prone to chance variation and should be interpreted as exploratory, serving primarily as a reference for future mechanistic work with larger cohorts, longitudinal follow-up, and integrated multi-omics profiling alongside host immune and barrier assessments. Second, we did not directly assess the functional potential of the microbiome or delineate the metabolic pathways through which it may influence barrier function or inflammatory responses. Future in vitro mechanistic experiments are planned to test these hypotheses.

## 5. Conclusions

Current research on human skin microbiota has predominantly focused on diseased skin or on facial and body sites, whereas systematic characterization of the scalp as a distinct ecological niche in healthy and subhealthy individuals remains limited. In this study, we specifically investigated young women with healthy or subhealthy scalps and integrated scalp typing, scalp sensitivity, lifestyle factors, and psychological stress to comprehensively describe the microbial composition and putative interactions at the scalp. Our findings suggest that scalp microecology may contribute to maintaining scalp health and shaping susceptibility to disease, providing a foundational reference for the prevention of suboptimal and pathological scalp conditions. Multifactor PERMANOVA further indicated that, among the physiological and lifestyle-related variables examined, scalp sensitivity is a relatively important factor of scalp microbiota structure, thereby offering a direction for future work on scalp physiology and pathology from the perspective of “microbiota–interaction–immune” networks.

## Figures and Tables

**Table 1:** Analysis of bacterial and fungal composition in samples at phylum level.

Bacterial Phyla	Relative Abundance	Fungi Phylum	Relative Abundance
Actinomycetota	65.32%	Basidiomycota	65.56%
Bacillota	33.78%	Ascomycota	35.35%

**Table 2:** Multifactor PERMANOVA analysis of sensitivity level, TEWL, sebum level, sleep, diet.

Factor	R^2^	Pr (>F)
Sensitivity Level	0.105	0.083
TEWL	0.031	0.151
Seb	0.001	0.837
Diet	0.005	0.637
Sleep	0.000692	0.91

## Data Availability

The data that support the findings of this study are available from the corresponding author on reasonable request.
